# Association between serum irisin concentrations and sarcopenia in patients with liver cirrhosis: a cross-sectional study

**DOI:** 10.1038/s41598-020-73176-z

**Published:** 2020-09-30

**Authors:** Mingyuan Zhao, Xiaoshuang Zhou, Chengying Yuan, Rongshan Li, Yuehong Ma, Xiaoxian Tang

**Affiliations:** 1 Department of Internal Medicine, Shanxi Provincial People’s Hospital Affiliated to Shanxi Medical University, Taiyuan, 030012 China; 2 Department of Radiology, Shanxi Provincial People’s Hospital Affiliated to Shanxi Medical University, Taiyuan, 030012 China

**Keywords:** Biomarkers, Diseases, Risk factors, Signs and symptoms

## Abstract

Sarcopenia is an independent predictor of mortality in patients with liver cirrhosis. However, evidence has emerged that skeletal muscles mediate their protective effect against sarcopenia by secreting myokines. Therefore, we investigated whether irisin was associated with sarcopenia in patients with liver cirrhosis. This was an observational cross-sectional study of data collected from 187 cirrhotic patients. Sarcopenia was defined by computed tomography (CT) scans using specific cutoffs of the 3rd lumbar vertebra skeletal muscle index (L_3_ SMI). Morning irisin levels were obtained in all patients. Of the 187 patients, sarcopenia was noted in 73 (39%). Irisin concentrations were lower in sarcopenic patients (32.40 pg/ml [interquartile range (IQR): 18.70, 121.26], *p* < 0.001) than in nonsarcopenic patients. There was a weak correlation between L_3_ SMI and irisin levels (*r* = 0.516, *p* < 0.001). Multivariable regression analysis including L_3_ SMI, body mass index (BMI), very-low-density lipoprotein (VLDL)-cholesterol, aspartate aminotransferase (AST), adiponectin, and irisin levels showed that L_3_ SMI (odds ratio [*OR*] = 0.915, *p* = 0.023), adiponectin levels (*OR* = 1.074, *p* = 0.014), irisin levels (*OR* = 0.993, *p* < 0.001) and BMI (*OR* = 0.456, *p* = 0.004) were independently associated with sarcopenia. Irisin levels are associated with sarcopenia in patients with liver cirrhosis. This paper addresses a gap in the literature and facilitates the future transition into clinical treatment.

## Introduction

Sarcopenia is a syndrome characterized by progressive and generalized loss of skeletal muscle mass and strength and has been shown to be prevalent in adults with liver cirrhosis. It is an important predictor of mortality in patients with liver cirrhosis and associated with a higher rate of infection and longer hospital stay, hepatic encephalopathy, poor quality of life, and increased healthcare cost^1^. The prevalence of sarcopenia in patients with cirrhosis varies, with reports ranging from 30 to 70%^2^. Some of the most important contributors to muscle wasting in cirrhosis are protein energy malnutrition, protein synthesis and breakdown, reactive oxygen species and inflammatory cytokines^3^. In addition to these factors, irisin could be a contributor^4^.

Irisin, a muscle-secreted protein, is released into the circulation by cleavage of fibronectin type III domain-containing protein 5 (FNDC5). Some researchers have suggested that irisin is very important as its physiological effects include reversing visceral obesity and improving glucose profiles^5^. Previous studies have found that the expression of irisin was detected immunohistochemically in hepatocytes, Kupffer cells, and sinusoidal endothelial cells^6,7^. An in vivo study showed severe fatty degeneration of the liver with impaired autophagy and fatty acid oxidation in FNDC5 knockout mice^8^. Since its identification in 2012, irisin has been suggested to play a favorable role in the context of metabolic diseases, including obesity, type 2 diabetes mellitus (T2DM), lipid metabolism and cardiovascular disease, nonalcoholic fatty liver disease (NAFLD), polycystic ovary syndrome, and metabolic bone diseases^9^. Despite fundamental etiopathogenetic differences between primary biliary cholangitis, NAFLD and viral cirrhosis, it is interesting to explore potential common links in sarcopenia with liver cirrhosis. Irisin is a promyogenic factor that induces myogenesis and mitochondrial biogenesis and protects against muscle atrophy. Increased in vivo irisin levels have been demonstrated to promote cell proliferation in various cell types^10–13^. However, to the best of our knowledge, the association between serum irisin concentrations and sarcopenia in patients with cirrhosis is unknown. There seems therefore to be a pressing need for clarifying these issues. In this study, we investigated the association of serum irisin concentrations with sarcopenia in patients with cirrhosis.

## Material and methods

### Study design

We conducted an observational cross-sectional study.

### Participants

Between January 2018 and January 2019, 187 cases of patients with liver cirrhosis were retrospectively reviewed in Shanxi Provincial People's Hospital (Shanxi, China). The inclusion criteria were patients who were 18 years of age or older, were nonpregnant, and provided written informed consent; the exclusion criteria included the presence of end-stage malignant diseases, acute generalized inflammation, acute infectious disease, history of drug abuse, renal insufficiency or chronic kidney disease. The study was approved by the local ethics committee, and all study participants gave written informed consent before taking part in the study.

The diagnostic criteria for liver cirrhosis were as follows: a history consistent with chronic liver disease, biochemical tests (albumin, prothrombin time, international normalized ratio (INR), total bilirubin, white blood cells, platelet count, transaminases, circulating triglycerides, very-low-density lipoprotein (VLDL), glucose, insulin, and hepatitis viruses), clinical features including clinical findings of decompensation of liver function (jaundice, malnutrition, spider angiomata, gynecomastia) and portal hypertension (varices, ascites, splenomegaly, hypersplenism), or a documented complication of chronic liver disease (esophageal variceal bleeding, hepatic encephalopathy) and/or imaging consistent with cirrhosis (presence or absence of varices, deformity of the liver or ascites) and/or liver biopsies (pseudolobuli formation).

### Outcome measures

#### Child–Pugh score

The Child–Pugh score^14^ is based on serum bilirubin and albumin, presence or absence of ascites, presence or absence of neurological disorder, and prothrombin time. The score values range from 5 to 15 points, as shown in Table 1 and are classified from A to C (class A, 5 to 6 points; B, 7 to 9 and C, 10 to 15 points).

**Table 1:** Child–Pugh score^14^.

Cirrhosis-Child–Pugh classification factor	1 point	2 points	3 points
Serum bilirubin µmol/l (mg/dl)	< 34 (< 2.0)	34–51 (2.0–3.0)	> 51 (> 3.0)
Serum albumin g/l (g/dl)	> 35 (> 3.5)	30–35 (3.0–3.5)	< 30 (< 3.0)
Ascites	None	Easily controlled	Poorly controlled
Neurological alterations	None	Minimum	Advanced coma
Prothrombin time (too slowly in seconds) *INR	0–4 < 1.7	4–6 1.7–2.3	> 6 > 2.3

*INR: international normalized ratio.

#### Measurement of skeletal muscle index (SMI) in our study

Hand grip strength was examined using a dynamometer (Daiyu,ShangHai) with three repetitions for each hand. The average of the three measures was recorded. The cutoff points for grip strength were 26 kg for men and 18 kg for women^3^. The highest value recorded for either hand was included in our study.

SMI is the most commonly used measure for defining sarcopenia. Based on the current guidelines, SMI is defined as skeletal muscle area (SMA)/height squared (m^2^), where SMA is measured using computed tomography (CT), and sarcopenia in patients with liver cirrhosis is less than 52.4 cm^2^/m^2^ for men and less than 38.5 cm^2^/m^2^ for women^3^. SMI was measured at the third lumbar vertebra (L_3_) measurement level. SMI was measured according to the current guidelines^3^. CT was performed on each patient to confirm the presence of sarcopenia in patients with liver cirrhosis. CT was independently evaluated by two experienced radiologists.

#### Blood collection and serologic assays

In all fasting subjects, a 5-ml blood sample was taken from the elbow vein. This sample was centrifuged for 15 min at 2,500 revolutions per minute, and then the serum was divided into 2 parts and frozen at -80 °C, where it was stored in the freezer until laboratory tests were performed.

Routine biochemical parameters, including albumin, creatinine, prothrombin time, INR, alanine aminotransferase (ALT), AST, total bilirubin, platelet count, total cholesterol, triglycerides, VLDL, and glucose, were measured for all patients according to standard methods in a routine clinical laboratory. Serum insulin, IL-6 and TNF-α (Phoenix Pharmaceuticals, Burlingame, CA, USA) were measured using enzyme-linked immunosorbent assays (ELISAs). The second serum sample obtained from the blood taken from the subjects was used to determine the concentration of irisin and adiponectin. Serum levels of irisin were determined using a commercial irisin ELISA kit according to the manufacturer’s instructions (Phoenix Pharmaceuticals, Burlingame, CA, USA). Sensitivity of the assay was 3 pg/ml, with a range of 15–1,200 pg/ml, intra-assay error of 9% and interassay error of 11%. The researcher who performed the ELISAs did not know the identity of the subjects.

Serum levels of adiponectin were determined using a commercial adiponectin ELISA kit according to the manufacturer's instructions. Sensitivity of the assay was 3 µg/ml, with a range of 15–1,200 µg/ml, intra-assay error of 9% and interassay error of 11%. The researcher who performed the ELISAs did not know the identity of the subjects.

The clinical and biochemical parameters were obtained within 1 week from the index CT scan used to determine the L_3_ SMI. The experimental protocol was approved by Shanxi Provincial People's Hospital. All methods were carried out in accordance with relevant guidelines and regulations.

Insulin resistance was calculated by the homeostasis model (HOMA-IR) using the following formula^15^: HOMA-IR = fasting insulin (mU/L) × plasma glucose (mmol/L)/22.5.

### Statistical analyses

Statistical analyses were performed according to our previously described methods^16^. The Shapiro–Wilk test was used to evaluate the distribution. Nonparametric tests were used except for age and plasma albumin. Differences in studied variables between groups were tested using the Mann–Whitney *U*-test and ANOVA range Kruskal–Wallis tests for independent groups. For bivariate analyses with continuous and categorical variables, we used the F2 test and the unpaired t test, respectively. Pearson’s correlation coefficient was used to evaluate the correlation between irisin levels and L_3_ SMI values. For multiple logistic regression analysis, we considered sarcopenia as the dependent variable. Irisin levels, BMI and L_3_ SMI values were entered as independent variables. Variables of interest plus variables with a *p* value < 0.1 in univariate analyses were included in multivariable regression analysis. We also used logistic regression to evaluate the association between sarcopenia as a dependent variable and irisin. Statistical analyses were performed using SPSS 18 (SPSS, Inc., Chicago, IL).

## Results

### Baseline characteristics in liver cirrhosis

Baseline characteristics in our study (n = 187; 102 males and 85 females; median age: 58 years, interquartile range [IQR]: 51, 66) are shown in Table 2. In terms of liver disease etiology, hepatitis B virus was shown in the majority (67.38%, 126/187). Irisin concentrations were significantly lower in the sarcopenia group (32.40 pg/ml, IQR: 18.70, 121.26) than in the no sarcopenia group (288.07 pg/ml, IQR: 176.42, 359.58) (*p* < 0.001). Adiponectin concentrations were significantly higher in the sarcopenia group (32.11 pg/ml, IQR: 23.82, 36.28) than in the no sarcopenia group (24.83 pg/ml, IQR: 18.38, 28.42) (*p* < 0.001). Furthermore, L3 SMI levels in the sarcopenia group were significantly lower (38.33 cm^2^/cm^2^, IQR: 34.78, 50.44) than in the no sarcopenia group (57.33 cm^2^/cm^2^, IQR: 49.01, -59.99) (*p* < 0.001; Table 2). We found that BMI was significantly decreased in the sarcopenia group (*p* < 0.001), as were triglycerides (*p* < 0.05). No differences were found between the two groups in the levels of albumin, ALT, AST, total bilirubin, platelet count, total cholesterol, VLDL-cholesterol, glucose, insulin, HOMA-IR, IL-6, TNF-α or prothrombin time (all *p* > 0.05; Table 2). There were no significant differences in the etiology (hepatitis B virus [HBV], hepatitis C virus [HCV], primary biliary cirrhosis [PBC] and others), gender (male and female), or Child–Pugh classification (A, B and C) between the two groups (all *p* > 0.05; Table 2).

**Table 2:** Features associated with sarcopenia in patients with cirrhosis.

Variables	All patients (*n* = 187)	No sarcopenia (*n* = 114)	Sarcopenia (*n* = 73)	*p* value
Age (years)	58 (51, 66)	56.5 (51, 66)	60 (52, 67.5)	0.402
Gender, male:female	102:85	67:47	35:38	0.97
HBV/HCV/PBC/others	126/17/28/16	75/11/16/12	51/6/12/4	0.636
Child–Pugh classification	40: 96: 51	32: 53: 29	8: 43: 22	0.20
BMI (kg/m^2^)	24.41 (22.99, 25.51)	25.12 (24.28, 28.12)	22.91 (22.15, 23.85)	< 0.001
L_3_ SMI (cm^2^/m^2^)	51.67 (40.22, 58.87)	57.33 (49.01, 59.99)	38.33 (34.78, 50.44)	< 0.001
Prothrombin time, INR	13.39 (12.16, 15.22)	13.41 (12.09, 15.28)	13.27 (12.15, 15.05)	0.96
Serum albumin (g/l)	29.81 (25.86, 34.51)	30.70 (26.5, 34.98)	29.33 (25.19, 33.53)	0.863
Total bilirubin (g/dl)	25.98 (17.49, 37.35)	26.05 (17.46, 36.61)	25.7 (17.56, 39.95)	0.752
ALT (IU/L)	27.36 (16.92, 42.51)	25.82 (15.72, 42.13)	30.95 (18.34, 43.97)	0.259
AST (IU/L)	33.23 (23.82, 51.21)	31.12 (23.26, 49.36)	34.74 (25.36, 59.8)	0.104
Platelet count (× 10^9^/mm^3^)	78 (60, 98)	78 (62, 98)	78 (59, 95)	0.421
Total cholesterol (mg/dl)	173.78 (156.86, 183.21)	173.82 (162.78, 183.45)	173.77 (147.2, 179.80)	0.636
Triglycerides (mg/dl)	106.86 (98.86, 133.14)	115.65 (99.75, 133.14)	105.73 (96.57, 132.14)	0.029
VLDL-cholesterol (mg/dl)	58.43 (53.21, 62.56)	58.81 (54.63, 62.39)	56.87 (52.17, 64.32)	0.073
Glucose (mg/dl)	113.23 (101.53, 124.5)	112.84 (101.26, 123.78)	113.34 (102.63, 128.59)	0.270
Insulin (IU/ml)	22.65 (12.88, 28.32)	22.73 (8.36, 27.97)	23.17 (14.97, 29.08)	0.211
HOMA-IR	3.90 (3.19, 5.69)	3.88 (3.08, 5.84)	3.98 (3.33, 5.40)	0.795
IL-6 (pg/ml)	11.57 (7.74, 22.65)	11.44 (7.82, 21.55)	13.45 (7.57, 25.22)	0.472
TNF-α (pg/ml)	17.89 (15.78, 19.45)	17.82 (14.85, 19.44)	17.92 (15.82, 19.59)	0.407
Adiponectin (µg/ml)	25.83 (21.07, 33.54)	24.83 (18.38, 28.42)	32.11 (23.82, 36.28)	< 0.001
Irisin (pg/ml)	185.69 (36.54, 321.11)	288.07 (176.42, 359.58)	32.40 (18.70, 121.26)	< 0.001

Data are expressed as the median value (interquartile range). Abbreviations: ALT: alanine aminotransferase. AST: aspartate aminotransferase. BMI: body mass index. L_3_ SMI: lumbar 3rd skeletal muscle index. HBV: hepatitis B virus. HCV: hepatitis C virus. HOMA-IR: homeostasis model assessment of insulin resistance. IL-6: interleukin-6. PBC: primary biliary cirrhosis. INR: international normalized ratio. TNF-α: tumor necrosis factor-α. VLDL: very-low-density lipoprotein.

### Analyses according to the Child–Pugh classification

#### Analyses of irisin according to the Child–Pugh classification

Based on the Child–Pugh classification, serum irisin concentrations in the no sarcopenia group were 264.15 pg/ml (IQR: 157.49, 358.95) for Child–Pugh A, 320.00 pg/ml (IQR: 210.39, 373.09) for Child–Pugh B, and 244.54 pg/ml (IQR: 143.18, 386.45) for Child–Pugh C. In the sarcopenia group, serum irisin concentrations were 149.41 pg/ml (IQR: 77.93, 189.65) for Child–Pugh A, 40.63 pg/ml (IQR: 18.84, 151.65) for Child–Pugh B, and 23.71 pg/ml (IQR: 16.48, 33.92) for Child–Pugh C (Table 3).

**Table 3:** Irisin concentrations stratified by Child–Pugh classification between the two groups.

	All patients	No sarcopenia group	Sarcopenia group	*z*-value	*p* value
Child–Pugh A (n = 40)	235.77 (132.93, 345.68)	264.15 (157.49, 358.95)	149.41 (77.93, 189.65)	2.401	0.016
Child–Pugh B (n = 96)	197.82 (36.58, 321.33)	320.00 (210.39, 373.09)	40.63 (18.84, 151.65)	6.590	< 0.001
Child–Pugh C (n = 51)	56.76 (25.01, 287.67)	244.54 (143.18, 386.45)	23.71 (16.48, 33.92)	5.249	< 0.001

Data are expressed as the median value (interquartile range).

**Figure 1:**
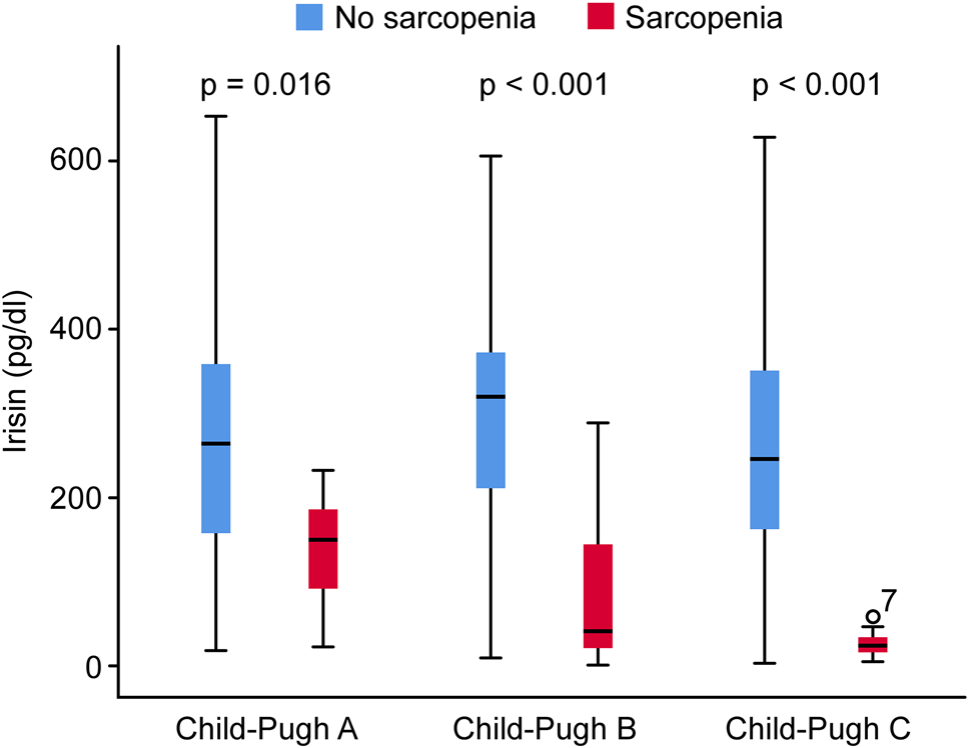
Boxplots of serum irisin concentrations in the no sarcopenia and sarcopenia groups of patients with liver cirrhosis, according to Child–Pugh classification. *p* values:* p* = 0.016 (no sarcopenia group vs. sarcopenia group for Child–Pugh A), *p* < 0.001 (no sarcopenia group vs. sarcopenia group for Child–Pugh B), *p* < 0.001 (no sarcopenia group vs. sarcopenia group for Child–Pugh C).

Nonparametric tests revealed significant differences between the no sarcopenia and sarcopenia groups for irisin levels in the Child–Pugh A (*Z* = 2.401, *p* = 0.016), Child–Pugh B (*Z* = 6.590, *p* < 0.001) and Child–Pugh C (*Z* = 5.249, *p* < 0.001) classes (Fig. 1; Table 3).

#### Analyses of adiponectin according to the Child–Pugh classification

Based on the Child–Pugh classification, serum adiponectin concentrations in the no sarcopenia group were 18.36 µg/ml (IQR: 16.48, 24.86) for Child–Pugh A, 24.89 µg/ml (IQR: 19.88, 26.73) for Child–Pugh B, and 33.54 µg/ml (IQR: 25.09, 37.55) for Child–Pugh C. In the sarcopenia group, serum adiponectin concentrations were 25.29 µg/ml (IQR: 21.58, 30.94) for Child–Pugh A, 27.82 µg/ml (IQR: 22.89, 36.12) for Child–Pugh B, and 34.65 µg/ml (IQR: 32.62, 38.59) for Child–Pugh C (Table 4).

**Table 4:** Adiponectin concentrations stratified by Child–Pugh classification between the two groups.

	All patients	No sarcopenia group	Sarcopenia group	*z*-value	*p* value
Child–Pugh A (n = 40)	19.19 (16.67, 25.66)	18.36 (16.48, 24.86)	25.29 (21.58, 30.94)	2.096	0.036
Child–Pugh B (n = 96)	25.46 (21.15, 29.47)	24.89 (19.88, 26.73)	27.82 (22.89, 36.12)	3.021	0.003
Child–Pugh C (n = 51)	34.45 (30.13, 37.56)	33.54 (25.09, 37.55)	34.65 (32.62, 38.59)	1.445	0.148

Data are expressed as the median value (interquartile range).

**Figure 2:**
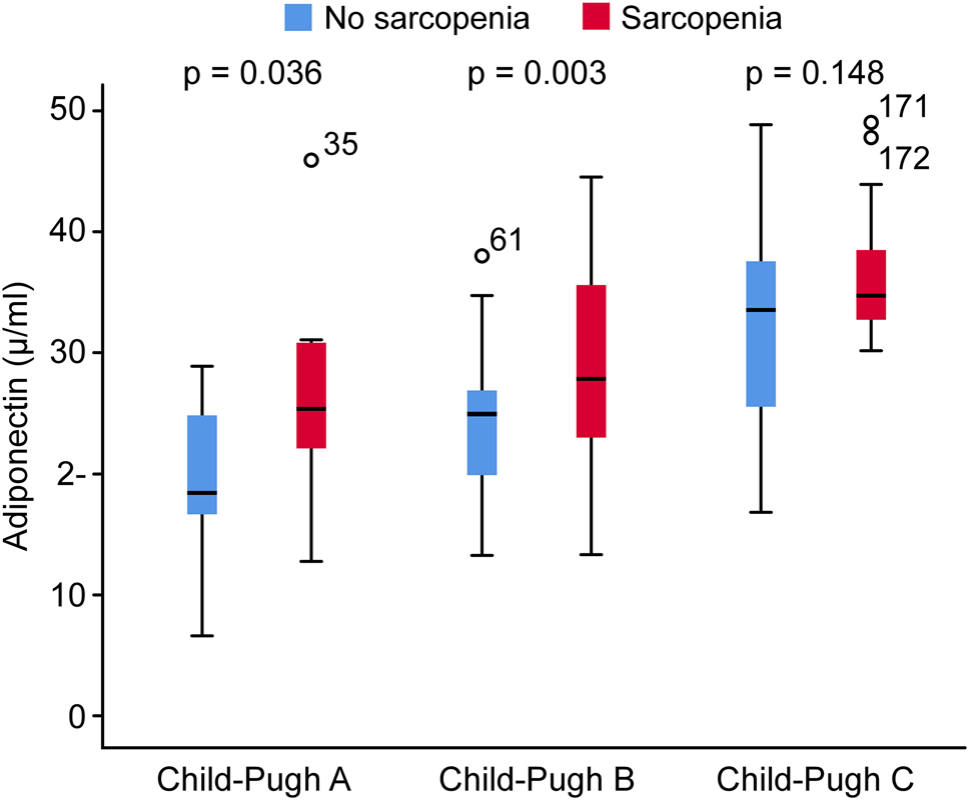
Boxplots of serum adiponectin concentrations in the no sarcopenia and sarcopenia groups with liver cirrhosis, according to Child–Pugh classification. *P* values:* p* = 0.036 (no sarcopenia group vs. sarcopenia group for Child–Pugh A), *p* = 0.003 (no sarcopenia group vs. sarcopenia group for Child–Pugh B), *p* = 0.148 (no sarcopenia group vs. sarcopenia group for Child–Pugh C).

Nonparametric tests revealed significant differences between the no sarcopenia and sarcopenia groups for adiponectin levels in the Child–Pugh A (Z = 2.096, *p* = 0.036), Child–Pugh B (Z = 3.021, *p* = 0.003) and Child–Pugh C (Z = 1.445, *p* = 0.148) classes (Fig. 2; Table 4).

### Features associated with sarcopenia in patients with cirrhosis by multivariable regression analysis

In patients with cirrhosis, univariate analyses identified three factors to be significantly associated with the presence of sarcopenia: L_3_ SMI (*p* < 0.001), BMI (*p* < 0.001), and serum adiponectin and serum irisin concentrations (*p* < 0.001) (Table 5). Variables with a *p* value < 0.1 in univariate analyses were entered in the multivariable regression analysis, including AST, triglycerides and VLDL.

**Table 5:** Features associated with sarcopenia in patients with cirrhosis by multivariable regression analysis.

	Variable analyses			Multivariate analysis		
	Hazard ratio	95% CI	*p* value	Hazard ratio	95% CI	*p* value
Age (year)	1.011	0.985–1.038	0.400			
Gender, male/female	1.548	0.857–2.796	0.148			
HBV/HCV/PBC/Others						
BMI (kg/m^2^)	0.267	0.185–0.387	< 0.001	0.456	0.267–0.779	0.004
L_3_ SMI (cm^2^/m^2^)	0.838	0.799–0.878	< 0.001	0.915	0.848–0.988	0.023
Prothrombin time, INR	1.030	0.930–1.140	0.570			
ALT	1.003	0.998–1.008	0.189			
AST	1.006	0.999–1.014	0.095	1.008	0.995–1.020	0.249
Serum albumin (g/dl)	0.973	0.929–1.019	0.243			
Total bilirubin (g/dl)	1.004	0.995–1.013	0.410			
Platelet count (× 10^9^/mm^3^)	0.997	0.990–1.004	0.446			
Total cholesterol (mg/dl)	0.966	0.991–1.002	0.161			
Triglycerides (mg/dl)	0.988	0.974–1.001	0.081			
VLDL-cholesterol (mg/dl)	0.960	0.916–1.008	0.100	0.968	0.903–1.037	0.353
Glucose (mg/dl)	1.004	0.989–1.020	0.578			
Insulin (IU/ml)	1.018	0.989–1.048	0.218			
HOMA-IR	0.972	0.841–1.125	0.705			
IL-6 (pg/ml)	1.004	0.992–1.015	0.540			
TNF-α (pg/ml)	1.049	0.972–1.132	0.219			
Adiponectin (µg/ml)	1.098	1.054–1.144	< 0.001	1.074	1.014–1.138	0.014
Irisin (pg/ml)	0.986	0.982–0.990	< 0.001	0.993	0.990–0.997	< 0.001

Data are expressed as the median value (interquartile range). Abbreviations: ALT: alanine aminotransferase. AST: aspartate aminotransferase. BMI: body mass index. L_3_ SMI: lumbar 3rd skeletal muscle index. HBV: hepatitis B virus. HCV: hepatitis C virus. HOMA-IR: homeostasis model assessment of insulin resistance. IL-6: interleukin-6. PBC: primary biliary cirrhosis. INR: international normalized ratio. TNF-α: tumor necrosis factor-α. VLDL: very-low-density lipoprotein.

Multivariate analysis for the six factors showed that BMI (*p* = 0.004), L_3_ SMI (cm^2^/m^2^) (*p* = 0.023), adiponectin levels (*p* = 0.014) and irisin levels (*p* < 0.001) were significant factors linked to the presence of sarcopenia (Table 5). Hazard ratios (HRs) and 95% confidence intervals (CIs) for these variables are listed in Table 5.

### Correlations between serum irisin concentrations and clinical and biochemical variables at baseline in patient liver cirrhosis

**Figure 3:**
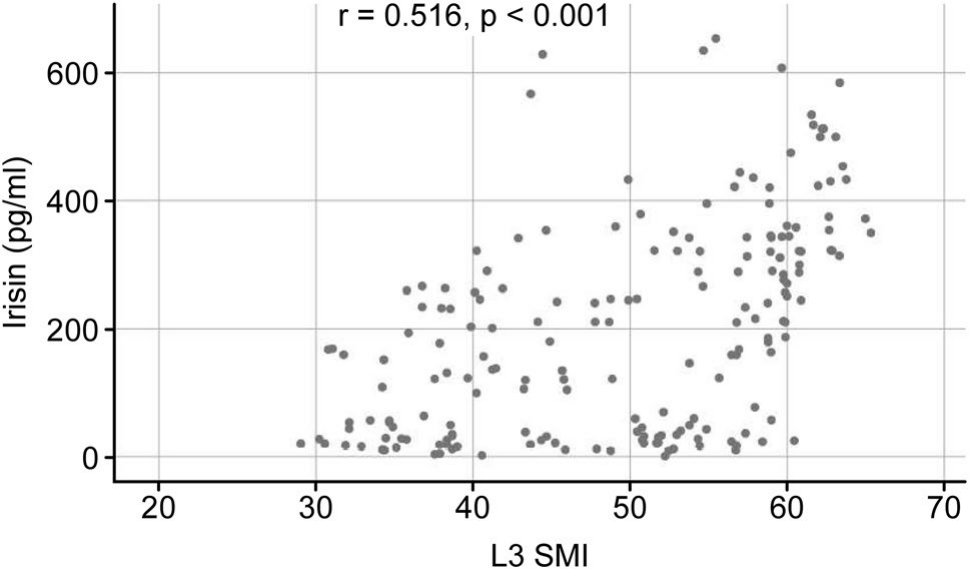
Correlation between serum irisin concentrations and L3 SMI values at baseline in patients with liver cirrhosis (r = 0.516, *p* < 0.001).

Serum irisin concentrations in the patients with liver cirrhosis were positively associated with L_3_ SMI values (*r* = 0.516, *p* < 0.001) and with BMI (*r* = 0.466, *p* < 0.001). In contrast, circulating irisin concentrations *did *not depend on age, gender, etiology, Child–Pugh classification, prothrombin time, total bilirubin, platelet count or total cholesterol (Fig. 3; Table 6).

**Table 6:** Correlations between serum irisin concentrations and clinical and biochemical variables at baseline in patients with liver cirrhosis.

Variables	R (*n* = 187)	*p* value
Age (years)	−0.12	0.87
Gender, male/female	0.108	0.139
Child–Pugh classification	−0.169	0.021
BMI (kg/m^2^)	0.466	< 0.001
L_3_ SMI (cm^2^/m^2^)	0.516	< 0.001
Prothrombin time, INR	−0.091	0.216
Serum albumin (g/l)	0.046	0.528
Total bilirubin (g/dl)	−0.089	0.604
Platelet count (× 10^9^/mm^3^)	0.135	0.451
Total cholesterol (mg/dl)	0.087	0.236
Triglycerides (mg/dl)	0.025	0.738
VLDL (mg/dl)	0.017	0.814
Insulin (IU/ml)	−0.044	0.554
HOMA-IR	−0.016	0.827
IL-6 (pg/ml)	0.032	0.661
TNF-α (pg/ml)	0.033	0.651
Adiponectin (µg/L)	−0.281	< 0.001

Bold values indicate statistical significance (*p* < 0.05).

### Correlations between serum adiponectin concentrations and clinical and biochemical variables at baseline in patients with liver cirrhosis

Serum adiponectin concentrations in the patients with liver cirrhosis were positively associated with Child–Pugh classification (*r* = 0.544, *p* < 0.001). In contrast, circulating adiponectin concentrations were not dependent on age, prothrombin time, total bilirubin, ALT, AST, L3 SMI, BMI, total cholesterol, triglycerides, VLDL-cholesterol, HOMA-IR, IL6, TNF-α and irisin (Table 7).

**Table 7:** Correlations between serum adiponectin concentrations and clinical and biochemical variables at baseline in patients with liver cirrhosis.

Variables	R (n = 187)	*p* value
Age (years)	−0.060	0.413
Child–Pugh classification	0.544	< *0.001*
BMI (kg/m^2^)	−0.140	0.056
L3 SMI (cm^2^/m^2^)	−0.237	0.001
Prothrombin time, INR	−0.077	0.292
Serum albumin (g/l)	−0.053	0.141
Total bilirubin (g/dl)	−0.189	0.009
Platelet count (× 109/mm3)	−0.070	0.344
Total cholesterol (mg/dl)	−0.085	0.249
Triglycerides (mg/dl)	−0.088	0.232
VLDL (mg/dl)	−0.27	0.712
HOMA-IR	0.107	0.143
IL-6 (pg/ml)	−0.330	0.657
TNF-α (pg/ml)	0.068	0.355
Irisin (pg/ml)	−0.281	< *0.001*

Bold values indicate statistical significance (*p* < 0.05).

## Discussion

The present study describes lower irisin concentrations in sarcopenic patients with cirrhosis than in nonsarcopenic patients with cirrhosis. In contrast, adiponectin levels were higher in sarcopenic patients with cirrhosis. We demonstrated for the first time that irisin was an independent parameter associated with sarcopenia in patients with liver cirrhosis. Associations between irisin and sarcopenia with some chronic diseases have been reported, including sarcopenic obesity, sarcopenia in postmenopausal women, sarcopenia in dialysis patients, and sarcopenia in those with myotonic dystrophies^17–20^.

To date, studies have long been aware that there is an association between liver disease and reductions in skeletal muscle mass. A large number of basic and clinical studies have been carried out in this field^3^. It is no simple task to provide a reason for this complicated phenomenon in sarcopenia, which involves many factors. Irisin is believed to play a role in metabolic diseases, aging, inflammation and neurogenesis. However, the plasma concentrations of irisin are closely correlated with several factors, such as diet, obesity, exercise, pharmacological agents and different pathological conditions. In our study, irisin concentrations were lower in sarcopenic patients with cirrhosis than in nonsarcopenic patients with cirrhosis (32.40 pg/ml [IQR: 18.70, 121.26] *vs*. 288.07 pg/ml [IQR: 176.42, 359.58], *p* < 0.001). It is not clear whether the lower irisin concentrations in sarcopenia patients with liver cirrhosis is caused by muscle wasting or sarcopenia is caused by the lower irisin levels. Irisin is secreted mainly by muscle and has been detected in various organs, such as the adipose tissue, brain, liver, kidney, and muscle itself. There is convincing evidence of a link between irisin and skeletal muscle mass. Building on research in postmenopausal women, irisin has been considered to be a sensitive molecular marker for muscle weakness and atrophy^20^. The decreased serum irisin concentrations can be used as a predictor for sarcopenia in postmenopausal women^19^. In fact, irisin has been proposed as a molecule that combines beneficial effects through restoration of bone and prevention of muscle wasting^21^. A recent study by Kalinkovich et al.^22^ indicated that irisin increased muscle mass and muscle hypertrophy. To date, few studies have investigated irisin in sarcopenia with liver cirrhosis. The mechanism by which irisin mediates the effect of sarcopenia on liver cirrhosis is still unknown.

We found a statistically positive correlation between irisin concentrations and L3 SMI values (r = 0.516, *p* < 0.001) and BMI (*r* = 0.466, *p* < 0.001) in all patients with liver cirrhosis. We did not find any statistically significant correlations between serologic assay results and irisin concentrations. Skeletal muscle makes up 40% of body weight and is mainly involved in mechanical activity requiring muscle fiber contractions. As sarcopenia is a frequent finding in patients with liver cirrhosis and irisin is a myocyte-secreted protein, it can be hypothesized that sarcopenia directly contributes to lower irisin levels. Our future study is to explore whether exogenous irisin could alleviate sarcopenia in patients with liver cirrhosis. Hanai et al.^23^ reported that skeletal muscle mass declined by 2.2% per year in 149 patients with cirrhosis. Based on the results of the Child–Pugh classification, the annual rate of decrease in skeletal muscle mass was 1.3% in Child–Pugh A patients, 3.5% in Child–Pugh B patients and 6.1% in Child–Pugh C patients^24^. Our results showed that plasma irisin concentrations were significantly lower in sarcopenic patients with liver cirrhosis when hepatic functional reserve gradually worsened. In the sarcopenic patients, the median (IQR) serum irisin concentrations were 149.41 pg/ml (IQR: 77.93, 189.65) for Child–Pugh A, 40.63 pg/ml (IQR: 18.84, 151.65) for Child–Pugh B, and 23.71 pg/ml (IQR: 16.48, 33.92) for Child–Pugh C (Table 3, Fig. 1). We speculate that the low irisin concentrations were caused by sarcopenia with liver cirrhosis. Clearly, these hypotheses need to be tested in future studies. These data suggest that irisin is not completely eliminated by the liver, and the clearance of irisin is achieved primarily through the renal system. Lv et al.^25^ showed that the metabolic clearance of irisin is achieved primarily through the hepatobiliary and renal systems. On the other hand, we should be aware that in the patients without sarcopenia, serum irisin concentrations were 264.15 pg/ml (IQR: 157.49, 358.95) for Child–Pugh A, 320.00 pg/ml (IQR: 210.39, 373.09) for Child–Pugh B, and 244.54 pg/ml (IQR: 143.18, 386.45) for Child–Pugh C (Table 3, Fig. 1). Irisin concentrations are not lower as hepatic functional reserve worsens (Table 3, Fig. 1). Irisin needs further study in the context of the pathology and physiology of liver cirrhosis.

Previous reports have shown that in addition to the role of adiponectin in carbohydrate and lipid metabolism, it has liver protection and anti-inflammatory actions that are also well known^26^. We found that adiponectin concentrations in sarcopenia patients with liver cirrhosis were significantly higher than those in the patients with liver cirrhosis without sarcopenia (32.11 µg/ml [IQR: 23.82, 36.28] vs 24.83 µg/ml [IQR: 18.38–28.42], *p* = 0.001). We found a statistically significant correlation between Child–Pugh classification and adiponectin concentrations in all patients with liver cirrhosis (r = 0.544, *p* < 0.001). This was in agreement with the findings of previous studies, which suggested that the increase in adiponectin concentrations in liver cirrhosis was positively correlated with the severity of liver injury and Child–Pugh scores^26,27^. The increase in adiponectin in liver cirrhosis is due to the difficulty in excreting adiponectin. We did not find a statistically significant correlation between L3 SMI values and adiponectin concentrations in all patients with liver cirrhosis (r = -0.237, *p* < 0.001). However, some researchers have observed associations between high serum adiponectin levels and low muscle cross-sectional areas, low muscle density, and poor function in the elderly population^26,27^. However, to date, there are few studies examining adiponectin in sarcopenic patients with liver cirrhosis.

We speculate that some patients with a Child–Pugh C classification, especially those with hepatic encephalopathy, gastrointestinal hemorrhage and a large amount of ascites, show a preserved level of strength during the grip strength test. It is not clear whether the grip strength test we used with these patients was influenced by subjective factors of the patients. Martínez Muñoz et al.^28^ found that hand grip strength seemed to be linked to irisin concentrations in overweight young women. Hand grip strength was reported to be a predictor for future development of type 2 diabetes mellitus^29^. Hand grip strength is considered to be a fast and simple test that is an indirect indicator of muscle strength^30^. We used grip strength in our study, but the next step is to study grip strength in patients with liver cirrhosis. Our study has some limitations. Although we have shown that there is a significant association between irisin concentrations and sarcopenia in patients with liver cirrhosis, we were unable to draw conclusions about causality because there is a lack of confidence in the randomized interventional study design. This type of investigation will not determine which factor, such as myostatin, adipokines, exercise, protein energy malnutrition and protein synthesis and breakdown, may play a role in this association. The impact of irisin on sarcopenic outcomes in patients with liver cirrhosis should be clarified in future studies. Muscle mass and muscle strength are important predictors of clinical outcomes in sarcopenic patients with liver cirrhosis, but it was unclear whether the grip strength test we used on these patients was influenced by subjective factors of the patients. We did not consider malnutrition, which represents a common complication in liver cirrhosis and affects patient outcome and recovery. Malnutrition may correlate with irisin levels. It is not clear how high adiponectin concentrations and low irisin concentrations affect skeletal muscle in patients with cirrhosis. Their mechanism of action and the relationship between irisin and adiponectin need to be further studied. Although this approach had limitations, we believe that this study provides useful information on the association between irisin concentrations and sarcopenia in patients with liver cirrhosis.

## Conclusions

In conclusion, the present study demonstrates for the first time that irisin concentrations are significantly associated with sarcopenia in patients with liver cirrhosis. Irisin levels can be an independent predictor of sarcopenia in patients with cirrhosis. To provide confirmation and examine possible mechanisms, additional studies are warranted.
